# Variation of MEMS Thin Film Device Parameters under the Influence of Thermal Stresses †

**DOI:** 10.3390/mi15101177

**Published:** 2024-09-24

**Authors:** Xiao Wen, Jinchuan Chen, Ruiwen Liu, Chunhua He, Qinwen Huang, Huihui Guo

**Affiliations:** 1 School of Information Engineering, Southwest University of Science and Technology (SWUST), 59 Qinglong Road, Mianyang 621010, China; wenxiao@mails.swust.edu.cn (X.W.); swustcjc@mails.swust.edu.cn (J.C.); 2 Science and Technology on Reliability Physics and Application Technology of Electronic Component Laboratory, China Electronic Product Reliability, and Environmental Testing Research Institute, Guangzhou 510610, China; 3 Institute of Microelectronics, Chinese Academy of Sciences, Beijing 100017, China; liuruiwen@ime.ac.cn; 4 School of Computer, Guangdong University of Technology, Guangzhou 510000, China; hechunhua@pku.edu.cn

**Keywords:** MEMS, thermal stress, thin film, deformation, resonant frequency, reliability

## Abstract

With the advancement of semiconductor manufacturing technology, thin film structures were widely used in MEMS devices. These films played critical roles in providing support, reinforcement, and insulation in MEMS devices. However, due to their microscopic dimensions, the sensitivity of their parameters and performance to thermal stress increased significantly. In this study, a Pirani gauge sample with a multilayer thin film structure was designed and fabricated. Based on this sample, finite element modeling analysis and thermal stress experiments were conducted. The finite element modeling analysis employed a combination of steady-state and transient methods to simulate the deformation and stress distribution of the device at room temperature (25 °C), low temperature (−55 °C), and high temperature (125 °C). The thermal stress test involved placing the sample in a temperature cycling chamber for temperature cycling tests. After the tests, the resonant frequency and surface deformation of the device were measured to quantitatively evaluate the impact of thermal stress on the deformation and resonant frequency parameters of the device. After the experiments, it was found that the clamped-end beams made of Pt were a stress concentration area. Additionally, the repetitive thermal load caused the cantilever beam to move cyclically in the Z direction. This movement altered the deformation of the film and the resonant frequency. The suspended film exhibited concavity, and the overall trend of the resonant frequency was downward. Over time, this could even lead to the fracture of the clamped-end beams. The variation of mechanical parameters derived from finite element simulations and experiments provided an important reference value for device design improvement and played a crucial role in enhancing the reliability of thin film devices.

## 1. Introduction

Micro-electromechanical systems (MEMS) technology achieved significant advancements in sensors and actuators by leveraging the scalability of integrated circuit manufacturing processes [1,2]. These advancements were reflected in dimensional accuracy, performance optimization, and cost-effectiveness. MEMS have been widely applied in various fields, including communications, automotive electronics, biomedical, and industrial control. Typically, the size of MEMS devices ranges from the nanoscale to the millimeter scale. As sensor sizes decrease and cost and energy consumption are reduced, designers must consider multiple factors in the design of MEMS devices, such as material selection, structural optimization, and environmental adaptability [3,4].

As a key component of MEMS devices, thin film structures exhibit high sensitivity to mechanical loads and environmental disturbances due to their small size [5]. The mechanical toughness of thin films is insufficient, making them susceptible to damage under external impact or localized stress concentration, such as crack initiation and propagation [6]. This can adversely affect the structural integrity and functionality of MEMS devices [7,8]. Additionally, in practical applications, MEMS devices often encounter extreme temperature environments, ranging from frigid conditions of around −50 °C to 120 °C [9]. Thin film materials, which are integral to these devices, respond particularly strongly to such temperature changes [10]. Due to the mismatch in the coefficient of thermal expansion (CET) between the thin film materials and the substrates or other films made of different materials, thermal stress is introduced at the interfaces of the material layers [11]. Such thermal stress can induce deformation in thin films, such as bending and warping, and may even lead to fracture [12,13,14]. This adversely affects the parameters and reliability of MEMS devices. Therefore, studying the impact of thermal stress on the parameters of MEMS thin film devices plays a crucial role in improving the performance and reliability of MEMS devices. Mohammad et al. explored the effects of thermal stress on the conductivity and microstructure of aluminum thin films on PET substrates [15]. By conducting isothermal aging and thermal cycling experiments, they analyzed the resistivity changes of films with different thicknesses under high temperatures and temperature fluctuations. The study found that increases in film thickness and heating time led to a rise in resistivity. Moreover, microstructural analysis revealed cracks and structural changes caused by thermal stress. These findings are crucial for improving the reliability and performance of thin film devices. Nathanael et al. investigated the in situ degradation monitoring of sputtered aluminum thin films on silicon cantilever beams using a nanoindenter (NI) inside a scanning electron microscope (SEM) [16]. They focused on the degradation behavior of thin film materials under thermal stress. By conducting high-cycle fatigue tests under isothermal mechanical loads and combining atomic force microscopy (AFM) and finite element modeling (FEM), they analyzed the relationship between surface roughness and equivalent plastic strain. This approach was used to evaluate changes in surface roughness and resistivity of MEMS device thin films under thermal stress conditions, as well as their reliability. Xinlin Peng et al. used COMSOL simulations to optimize the structure and perform mechanical analysis of MEMS thin film getter–heater units [17]. They focused on the stress and deformation behavior of cantilever structures and compared the performance of various cantilever designs. The symmetrical-shaped heater with the edge-center located cantilever model (II-ECLC model) was ultimately selected due to its minimal deformation under thermal stress. This model significantly enhanced the device’s resistance to interference in thermal stress environments.

This paper used COMSOL Multiphysics to model and simulate multilayer thin film structures [18]. It examined the thermal stress distribution and deformation of devices under extreme conditions. The results showed that the cantilever area and platinum metal resistors are the primary regions of stress concentration. Under alternating hot and cold thermal cycling conditions, the middle thin film section of the device exhibited repetitive upward and downward displacement. Repeated upward and downward displacements exacerbated the stress concentrations in the cantilever beams, a condition that may have been a key factor in the failure of the device. Additionally, through thermal stress testing, we measured the deformation and resonant frequency, along with other mechanical parameters, of the device after multiple cycles. The results showed that the concavity of the device’s thin film gradually increased, while the resonant frequency exhibited a dynamic decreasing trend. This may be due to a reduction in film stiffness. The results of this study provide important references for the design improvement of MEMS multilayer thin film devices. The study also identifies potential risk factors that could lead to failure, contributing to the enhancement of device stability and reliability.

## 2. Samples

**Figure 1:**
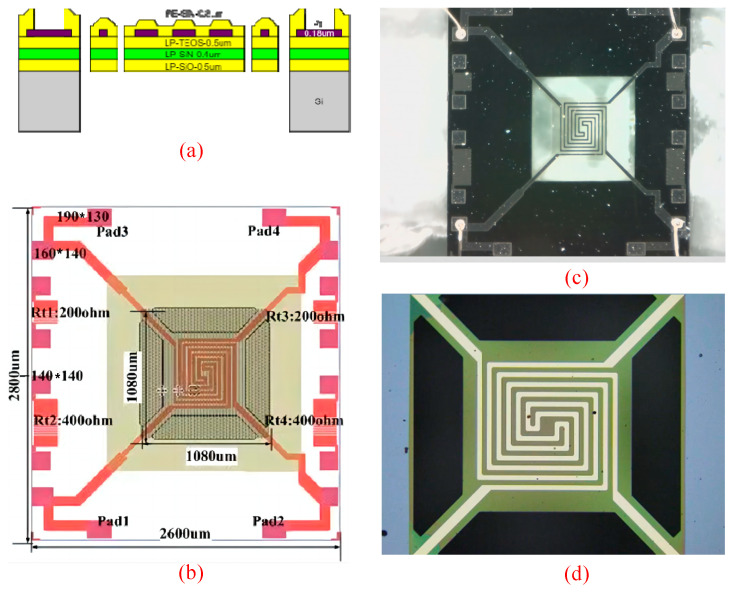
Pirani gauge with a multilayer composite thin film structure and a four-terminal clamped-end beam. (**a**) Side view of the sample; (**b**) overall top view of the sample; (**c**) physical picture under the optical microscope; (**d**) the physical picture under the profilometer.

The test sample is a Pirani gauge with a multilayer suspended composite thin film structure and a four-terminal clamped-end beam. It is based on the thermal conductivity principle and determines gas pressure by measuring the impact of gas on heat conduction. Each sample contains two platinum resistors. The Pirani gauge operates based on the thermal conductivity principle, determining gas pressure by measuring the effect of gas on heat conduction. In this system, each sample contains two platinum resistors. One platinum resistor typically acts as a heating element, which is heated by an electric current. As the density of gas molecules changes, the transfer of heat from the platinum resistor to the gas is affected. The temperature change of the heating element is then measured by another platinum resistor, enabling an indirect measurement of gas pressure. The main hierarchical structure of the sample, from bottom to top, consists of a silicon substrate, silicon dioxide film, silicon nitride film, silicon dioxide film, platinum metal film, and silicon nitride film (As shown in Figure 1a). During operation, when gas molecules collide with the surface film, they carry away heat from the film, causing a decrease in the film temperature. The built-in thermistors can measure temperature changes. The resistor positions are labeled as shown in Figure 1b and the parameters are shown in Table 1. Subsequently, through data processing, it can be compared with the thermal conductivity at known gas pressures to determine the gas pressure. Due to its multilayered thin-film structure and frequent variations in operating temperature, the Pirani gauge is more susceptible to thermal stress. Therefore, analyzing the impact of thermal stress on the device is of paramount importance.

LP in Figure 1a stands for LPCVD (Low-Pressure Chemical Vapor Deposition), and low pressure refers to the low pressure of the gas in the reaction chamber. To ensure deposition rate, the deposition temperature is typically adjusted to higher than 600 °C. During the LPCVD process, precursor gases are introduced into the reaction chamber at low pressure, where they chemically react with the substrate surface to form deposited material. The LPCVD process typically operates under high vacuum conditions. By controlling the pressure and flow rate of the reaction gases, it is possible to achieve control over deposition rates and ensure uniformity of the film. Due to the lower pressure and higher deposition temperature, LPCVD is suitable for producing high-quality, high-purity thin film materials such as polycrystalline silicon films.

PE stands for PECVD (Plasma-Enhanced Chemical Vapor Deposition). Plasma enhanced refers to the use of plasma to enhance the chemical reactions during the deposition process. One remarkable advantage of the PECVD process is its relatively low deposition temperature. During the PECVD process, an electric field or RF power is applied to activate the gas and form plasma. Electrons and ions in the plasma collide with gas molecules, generating excited states and dissociation reactions that facilitate the deposition process. Due to the involvement of plasma, PECVD processes enable film deposition at relatively low temperatures, typically below 300 °C. This makes PECVD suitable for temperature-sensitive materials on substrates such as organic films, nitrides, and oxides.

The LP-TEOS process involves generating SiO_2_ films through the low-pressure chemical vapor deposition of tetraethyl orthosilicate. Tetraethyl orthosilicate is a commonly used silicon source. It is introduced into the reaction chamber and chemically reacts with the substrate surface under specific deposition conditions, forming a SiO_2_ film. This process has the advantage of producing SiO_2_ films with better coverage and uniformity. The LP-TEOS process benefits from tetraethoxysilane, which offers a stable silicon source. Under low pressure, the deposition occurs uniformly, allowing the film to grow evenly on the substrate surface. A uniform film ensures consistent properties and performance across the entire substrate. This characteristic makes the resulting film widely used as a dielectric layer, isolation layer, and protective layer. The specific chemical reaction equation is as follows:

Si(OC_2_H_5_)_4_ + 2O_2_ → SiO_2_ + 4C_2_H_5_OH (1)

The heating resistor made of platinum is primarily utilized to generate a constant heat source, enabling the measurement of gas thermal conduction and indirect inference of gas pressure. The platinum heating resistor has several advantages: high strength, good linearity, simple manufacturing process, high yield, and excellent reliability. It is commonly used in research and daily production environments at temperatures ranging from −200 to 850 °C. Platinum’s chemical stability at room temperature makes it ideal for use in Pirani gauges. These gauges typically operate at temperatures between 100 °C and 400 °C and under low pressure. Under these conditions, platinum heating resistors do not undergo oxidation, ensuring stable resistance values and, consequently, the sensor’s reliability and stability.

## 3. Finite Element Analysis

**Figure 2:**
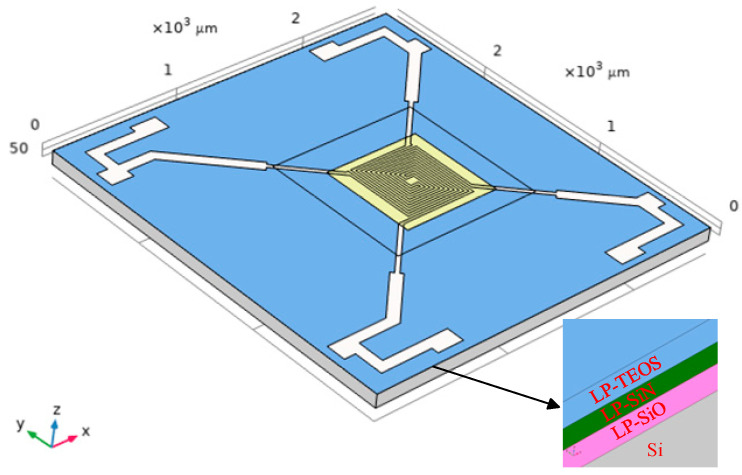
Finite element modeling of MEMS multilayer film structure Pirani gage.

The Pirani gauge has a multilayer thin film structure, and its working environment experiences significant temperature variations. Changes in thermal stress caused by these temperature fluctuations can severely impact the mechanical properties of the film-substrate structure. Excessive stress may lead to failure issues such as interface delamination, film bending, and stress concentration. These changes ultimately affect the reliability and stability of the measurement results of the Pirani gauge device. Meanwhile, the multilayer composite film structure of the four-terminal clamped-end beam, which is not attached to the substrate, will also undergo changes in mechanical properties under the influence of thermal stress, thereby affecting the device’s performance. To study the thermal stress of the multilayer composite film structure of the four-terminal clamped-end beam Pirani gauge, a finite element model of the device was created. A multiphysics thermal expansion simulation analysis was also conducted based on solid mechanics and heat transfer. In this model, the bottom surface of the device is set as a fixed constraint surface. The insulation holes present in the actual device were omitted from the model for simplification, as their influence is minimal in the context of thermal stress simulations without electrical current. The simplified finite element model is shown in Figure 2 and the relevant material parameters are given in Table 2.

Although residual stresses generated during the deposition process are known to exist, these stresses were not included in the simulation for two primary reasons. First, the focus of this study is on the stress and deformation behavior under actual operating conditions, such as thermal cycling and mechanical loading, which are critical for assessing the device’s reliability. Second, residual stresses from the deposition process are complex to model accurately, as they depend on various parameters like deposition technique and rate. Including these stresses could introduce uncertainties, and as a result, they were simplified or omitted in our analysis to better isolate the operational effects of thermal stress.

**Figure 3:**
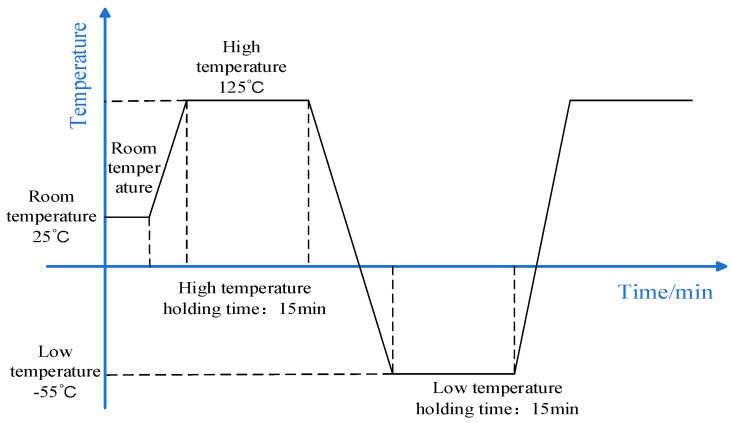
The external temperature function pw1(t).

In this simulation analysis, two main physical fields were included: solid mechanics and heat transfer in solids. The heat transfer in solids used a transient equation form primarily to simulate the heating process. Solid mechanics employed a stationary equation form, inheriting all the solutions from the previous step to calculate the solid mechanics. In the heat transfer in solids, an external temperature function pw1(t) was incorporated. The function simulated the temperature variations of a thermal shock test cycle. The initial temperature was set to room temperature to represent the device’s condition before the experiment. The external temperature function pw1(t) is shown in Figure 3:

The entire structure is subjected to both high and low temperatures depicted in Figure 3 to simulate the complete temperature cycling that the device would experience in practice. This ensures that the full range of temperature variations and their impact on the device are represented. Heater operation was not modeled, as the focus of this study is on the device’s thermal stress distribution and mechanical behavior, rather than on the heater’s operation. Adding heater dynamics would introduce unnecessary complexity without contributing to the analysis of stress and deformation.

**Figure 4:**
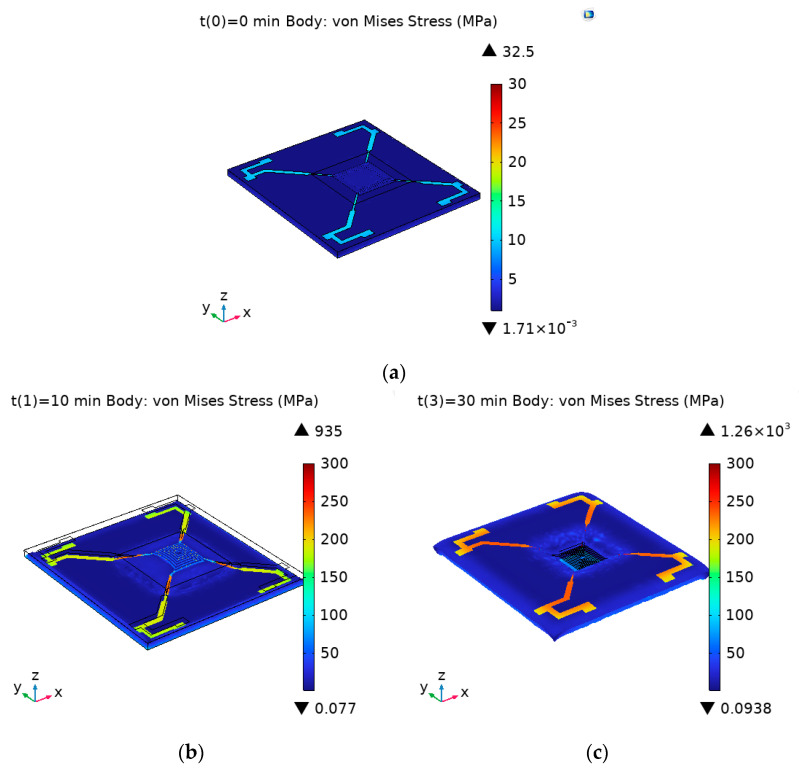
Surface deformation and stress distribution in three states of MEMS multilayer thin-film-structured Pirani gage. (**a**) The temperature is 25 °C; (**b**) the temperature is −55 °C; (**c**) the temperature is 125 °C.

**Figure 5:**
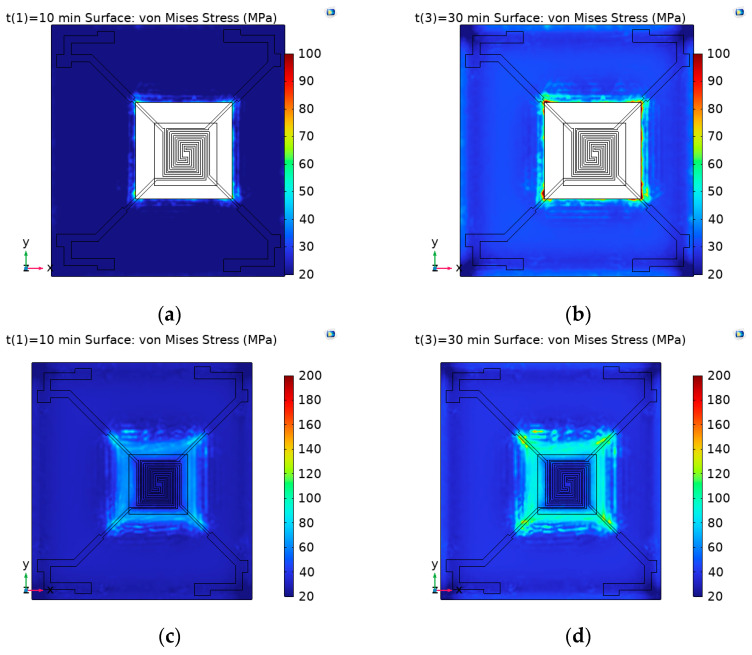

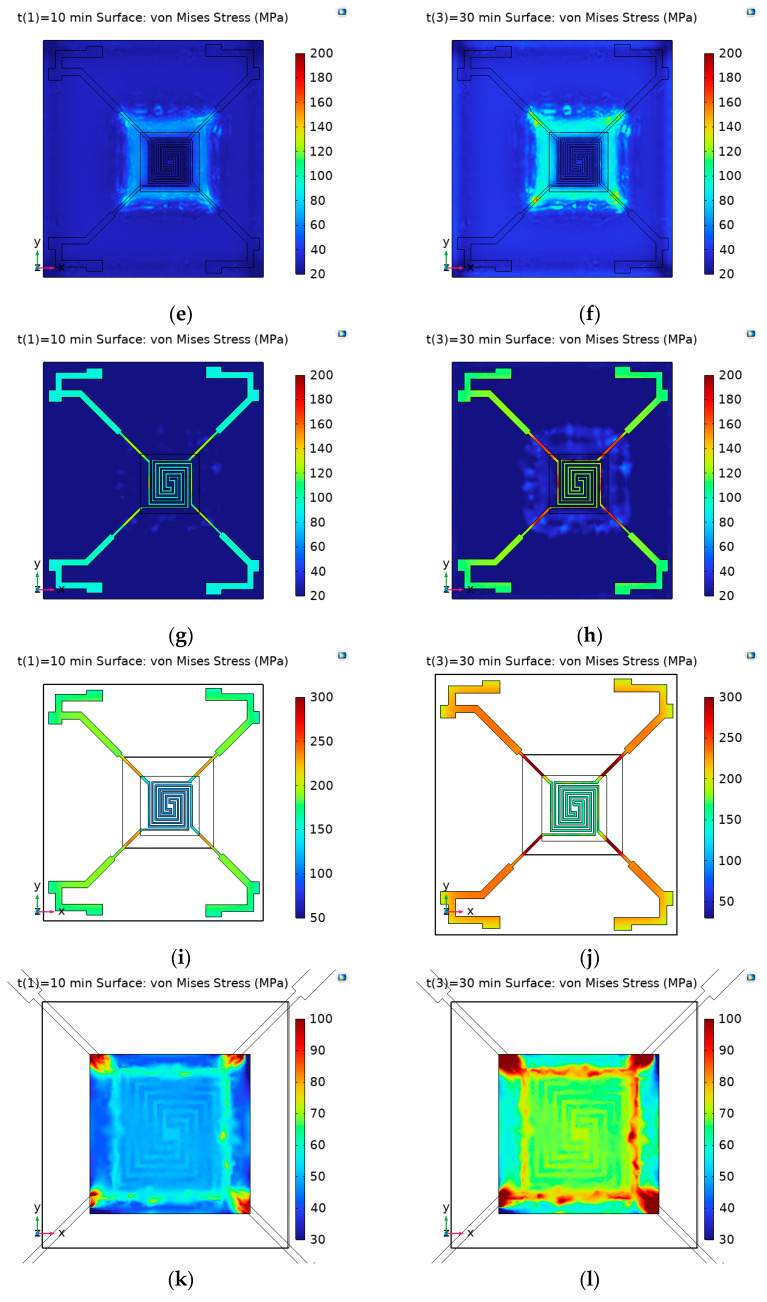
Stress distribution on the upper surface of each layer. (**a**,**c**,**e**,**g**,**i**,**k**) show the thermal stress distribution on the upper surface of the silicon substrate, silicon dioxide, silicon nitride, silicon dioxide, Pt, and silicon nitride layers, respectively, at low temperature; (**b**,**d**,**f**,**h**,**j**,**l**) show the corresponding thermal stress distribution at high temperature.

In the transient equation study, the output time and step size were set. The output time ranged from 0 min to 35 min, with a step size of 10 min. In other words, the heat transfer in solids analysis of the study target was sampled every 10 min over a 35 min period. In the extended study section of the stationary analysis, auxiliary scanning was set with time t as the scanning parameter. The same time step as in the transient analysis was used, thereby linking the transient and stationary analyses. The stress distribution in three different states is shown in Figure 4:

As shown in Figure 4, the effect of thermal stress on the device was mainly influenced by the temperature difference. In both high and low-temperature conditions, the morphology of the device changed significantly, and stress concentration areas appeared. By analyzing the thermal stress on each layer’s surface separately under high and low-temperature conditions, the results shown in Figure 5 were obtained. (a), (c), (e), (g), (i), and (k) denote the thermal stress distribution on the upper surface of the silicon substrate layer, silicon dioxide layer, silicon nitride layer, silicon dioxide layer, Pt layer, and silicon nitride layer, respectively, at low temperature; (b), (d), (f), (h), (j), and (l) then represent the thermal stress distribution on the upper surface of the corresponding layer at high temperature.

From the magnitude and distribution of stress on the top surface of each layer, it can be seen that the size of the thermal stress is related to the temperature difference, materials, and structure. Regarding temperature difference, the larger the difference, the greater the thermal stress. At the same high-temperature thermal stress, the maximum stress observed in silicon dioxide was approximately 150 MPa, which indicates it is the least affected by temperature changes. Silicon nitride experienced a higher maximum stress of approximately 180 MPa, showing that it is more affected by temperature fluctuations compared to SiO_2_. Platinum showed the highest thermal stress, with a maximum stress of approximately 300 MPa, making it the material most affected by temperature changes. Structurally, the clamped-end beam made of Pt material is a stress concentration area. The inner edge of the silicon substrate also shows significant stress concentration. The coverage of the top silicon nitride film can reduce the thermal stress on the Pt material to some extent, providing it with support and protection.

**Figure 6:**
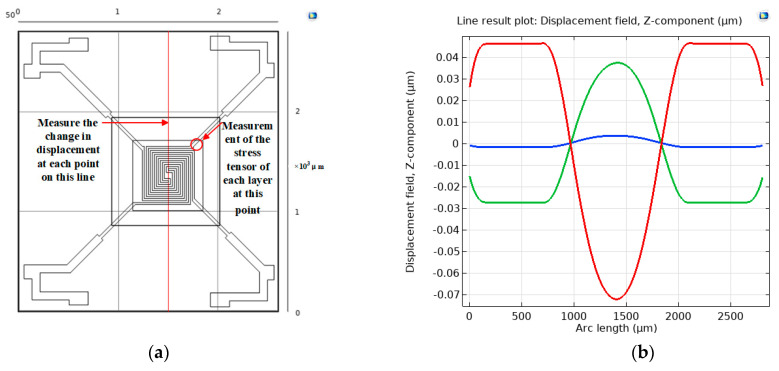
Simulation measurement of device surface deformation. (**a**) Selection of the measured part with an intercept line; (**b**) deformation of the measured part in three states.

To further investigate the stress distribution and variations in the multilayer thin-film structure under temperature cycling, three-dimensional cross-sectional lines were added to the data set in the results section. As shown in Figure 6a, a cross-sectional line was added to the device surface. Additionally, a longitudinal cross-sectional line was added at a point on the suspended clamped-end beams, passing through all the thin film materials, starting from the bottom and moving upwards. The added cross-sectional lines can be categorized into two types. One type was used for analyzing the surface deformation of the device. The other type was used for observing the stress in different layers along the same X and Y coordinates. The stress distribution in Figure 4 indicates significant stress variation in the beams; therefore, the detection point was chosen at this location. This area was also the most prone to fracture in the thin-film structure. Studying this location can aid in the failure analysis of the device. Adding a 1D plot group in the results section allowed for the observation of stress and deformation along the specified section line.

Figure 6b shows the deformation along a straight line passing through the center of the heating resistor on the LP-TEOS layer of the device surface. The figure represented surface deformation by displacements at various points along the line. For the analysis of thermal mismatch strain in substrate/film systems, the following theoretical approaches were commonly adopted:

Assuming two temperature states, $T_{d}$ was the unstressed state and $T_{r}$ was the room temperature, the thermal strain of the substrate

(2) $$\epsilon_{s}=-\alpha_{Ts}\Delta T$$

Among them,

(3) $$\Delta T=T_{d}-T_{r}$$

$\alpha_{Ts}$ was the coefficient of thermal expansion of the substrate material. Assuming that the film is not attached to the substrate, its thermal strain is represented as follows:

(4) $$\epsilon_{f,\;\mathrm{free}\;}=-\alpha_{Tf}\Delta T$$

In this equation, $\alpha_{Tf}$ represents the coefficient of thermal expansion of the film. When the film is attached to the substrate, its actual strain is equal to the strain of the substrate. The formula is as follows:

(5) $$\epsilon_{f,\;\mathrm{attached}\;}=-\alpha_{Ts}\Delta T$$

There existed an additional strain known as the thermal mismatch strain. This strain represented the difference between the actual strain of a film attached to a substrate and the strain of a film not attached to a substrate:

(6) $$\epsilon_{f,\;\mathrm{mismatch}\;}=\left(\alpha_{Tf}-\alpha_{Ts}\right)\Delta T$$

**Figure 7:**
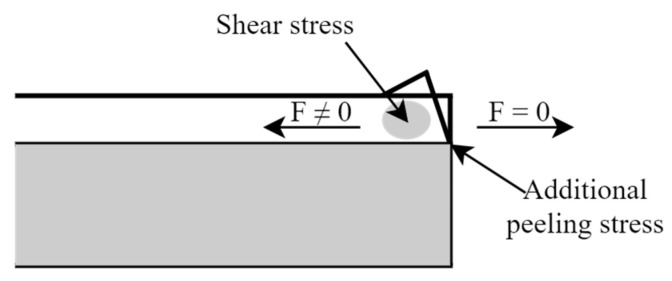
Schematic diagram of edge film warping by thermal stresses.

At the edge of the substrate/film system, there was a special situation. For example, when the device was exposed to a temperature of 125 °C, the film experienced tensile stress away from the edges. This resulted in a non-zero net in-plane force in the regions away from the edges. However, at the edges, which were free boundaries, the net in-plane force was zero. Since there was no in-plane force at the edges, the compression in the vertical direction was released. This caused the film to thicken and slightly bend inward. The principle of action is shown in Figure 7.

The simulation results revealed that at 30 min, there was an expansion trend, with a relative sinking observed in the central structural portion. The simulated temperature cycling experiment was conducted by alternately heating and cooling to simulate the cyclic loading and unloading of thermal stress in materials during use. This experimental method can release residual stresses in the device through the deformation and rebound processes of cyclic loading and unloading. The conditions of temperature cycling provided mechanisms such as structural changes within the material and the movement of dislocations and defects to reduce or eliminate residual stresses.

**Figure 8:**
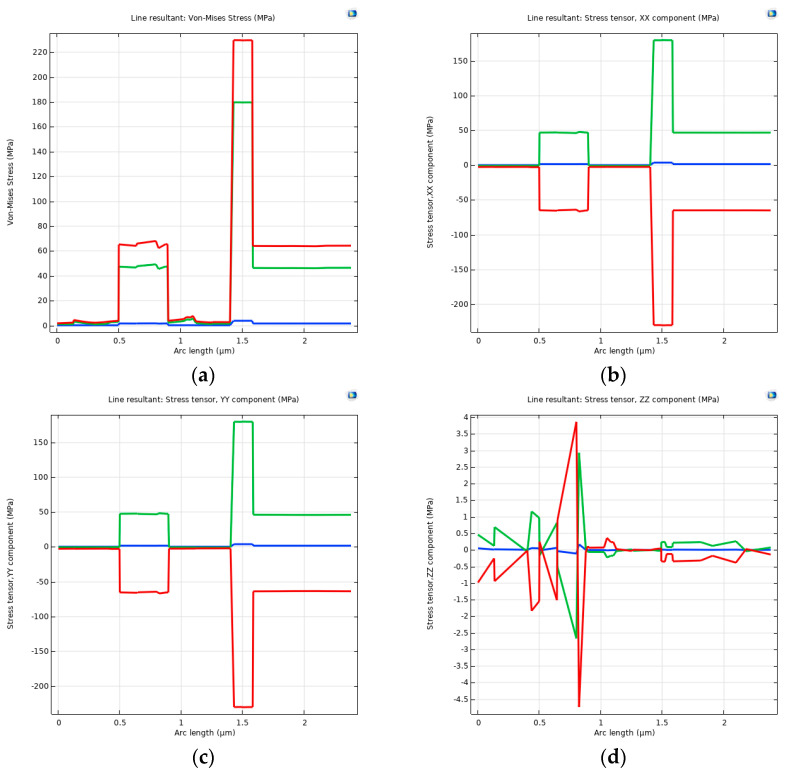
Stresses in films in layers below a point on a clamped-end beam (blue for 25 °C, green for −55 °C, and red for 125 °C). (**a**) Von Mises stresses; (**b**) stress tensor in XX direction; (**c**) stress tensor in YY direction; (**d**) stress tensor in ZZ direction.

Measurement of stress conditions across different layers at the same X and Y coordinates yielded the results shown in Figure 8a. The displayed stress was the von Mises stress, with the figure illustrating only the magnitude of the force. Figure 8b–d separately show the stress tensors in the XX, YY, and ZZ directions. It can be observed that the stress tensor is mainly concentrated in the XX and YY directions. The maximum stress in the ZZ direction was less than 4 MPa, which was two orders of magnitude lower than the stress tensor in the other two directions. Furthermore, the stresses were mainly concentrated on the LP-SiN layer, Pt layer, and PE-SiN layer.

## 4. Experiment

The experimental phase primarily involved subjecting samples to temperature cycling tests. These tests accelerated the release of stresses in the devices through cyclic loading and unloading deformation and rebound processes. Subsequently, the evolution of stresses was observed and analyzed. The content of the experiment mainly referred to the method 1010.1 in GJB 548B-2005 [19].

### 4.1. The Entire Experimental Process

**Figure 9:**
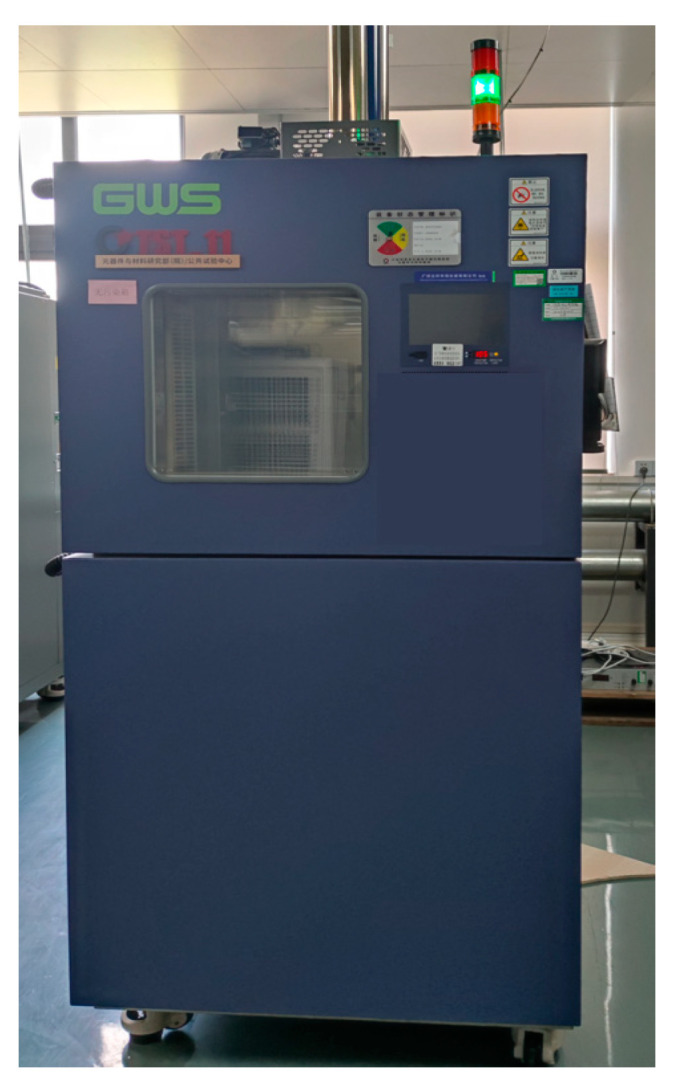
Temperature cycling test chamber.

The temperature cycling test chamber, sourced from GWS in Guangzhou, China, shown in Figure 9, is divided into two areas: the upper chamber is the high-temperature zone and the lower chamber is the low-temperature zone. The interior is equipped with a basket that can be moved up and down to quickly switch the position of the specimen between the high and low-temperature zones to achieve rapid changes in temperature. The device is able to simulate the performance of products under extreme temperature conditions and assess their ability to withstand heat, cold, and temperature shock.

The entire experimental process was conducted as follows: Initially, the resonant frequency and height difference of the samples were measured at room temperature. The samples were then placed in a temperature cycling chamber and exposed to conditions of 125 °C for high temperature and −55 °C for low temperature, with each temperature maintained for 15 min. After every 100 cycles, the samples were removed and measured for their resonant frequency and surface deformation (height difference) at room temperature. This process of temperature cycling and measurement was repeated until the samples either completely failed or the desired test results were achieved.

### 4.2. Resonance Frequency Test

**Figure 10:**
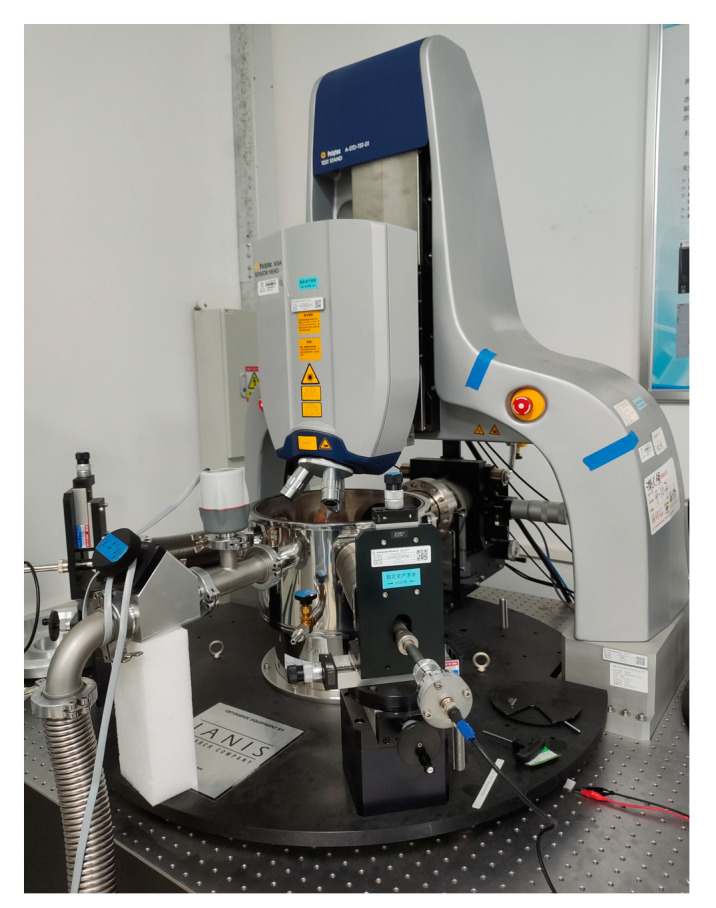
Polytec MSA-600-VD scanning laser vibration measurement and modal analysis system.

A theoretical model considering residual stress was established by Peng et al. [20]. The modal characteristics of MEMS resonator arrays were measured using laser Doppler and strobe observation methods. The resonant frequency test equipment is shown in Figure 10. The study showed that residual stress significantly affects the natural frequency of MEMS devices.

The modal measurements of the multilayer composite thin-film structure with four-terminal clamped-end beams were conducted using a Polytec MSA-600-VD scanning laser vibration measurement and modal analysis system, sourced from Polytec in Waldbronn, Germany. The shock-absorbing platform was opened and the piezoelectric plate was connected to the excitation signal of the test system. The samples were then adhered to the piezoelectric plate. The PSV 6.0 software was launched and the excitation frequency range was set from 1 kHz to 100 kHz, with a voltage value of 1 V. The test area was defined using laser scanning. The excitation signal was activated to perform a modal analysis of the MEMS device. After the analysis, the excitation signal was turned off and the frequency sweep results were saved.

### 4.3. Surface Deformation (Height Difference) Test

**Figure 11:**
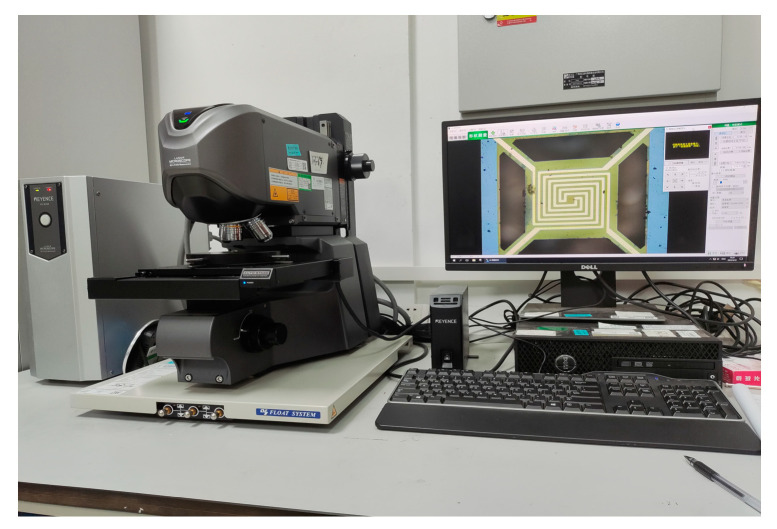
Keyence VK-X260K 3D optical profilometer.

During the alternating heating and cooling process, the stress in the device was released through the cyclic loading and unloading at high and low temperatures, causing deformation and rebound. The multilayer composite film structure of the four-terminal clamped beams underwent certain changes. To understand the evolution of residual stress, it was necessary to observe the surface deformation of the multilayer composite film structure of the four-end clamped beams. The KEYENCE VK-X260K 3D optical profilometer, sourced from KEYENCE Corporation in Osaka, Japan, was used for this experiment, and the steps for surface topography measurement were as follows: The shock-absorbing platform was activated, and the test samples were placed on the platform. Using a low magnification lens, the device was centered. The eyepiece was adjusted to 50×/0.45 and a reference height was established. The image stitching function was used to ensure that the resulting image included the required parts. The test was then initiated, a reference plane was selected, and the test area was defined. Finally, the test results were saved and organized. The test equipment is shown in Figure 11:

## 5. Result and Discussion

**Figure 12:**
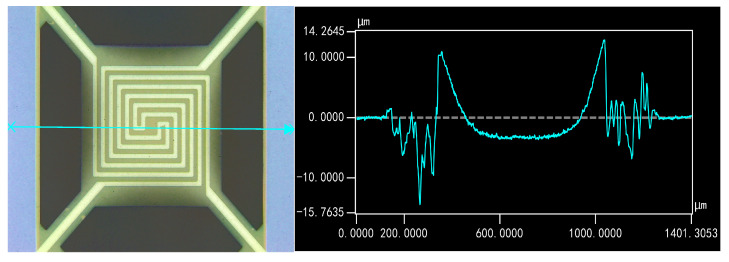
Intermediate surface morphology under the influence of residual stresses during deposition.

During the finite element modeling analysis of the device, the results in Figure 6 clearly showed that the material repeatedly deformed under temperature cycling. The part with the silicon substrate exhibited surface warping at the edges, while the multilayer composite film structure of the four-terminal clamped beams showed a tendency to concave. The test site selected for the experiment is shown in Figure 12:

In the actual device, there was an initial large deformation due to the thermal stress from the manufacturing process, which was not included in the simulation. Additionally, the black region in Figure 12 corresponds to insulation holes that are directly connected to the bottom substrate. The large height difference in this area caused distorted data from the profilometer, leading to the irregularities observed.

**Figure 13:**
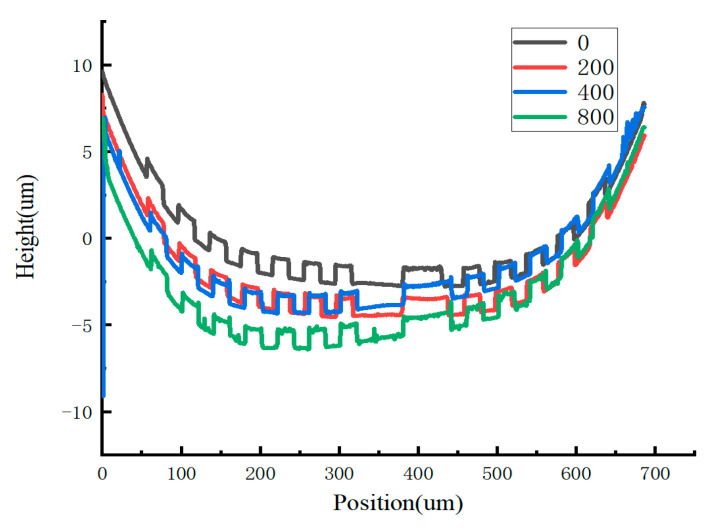
Trends in deformation of the multilayer composite film structure of the four-terminal clamped beams during temperature cycling experiments.

In this experiment, we focused on the surface deformation in the multilayer composite film structure of the four-terminal clamped beams, as this is closely related to the device’s failure. By using an optical profilometer at 50× magnification, we compared the effects of thermal stress effect on the central section of the device structure after different numbers of temperature cycles. It was observed that as the number of thermal cycling cycles increased, the multilayer composite film structure of the four-terminal clamped beams tended to become concave. High temperatures can cause atomic rearrangement, reducing internal defects and dislocations and leading to stress relaxation. The observed deformation could be the result of internal residual stress being redistributed or partially released due to cyclic thermal loading. The increase in deformation, as illustrated in Figure 13, reflects this redistribution of internal stresses through stress relaxation. The surface deformation observed in sample 2’s multilayer composite film structure further highlights the trend of relative concavity in this part of the device.

**Figure 14:**
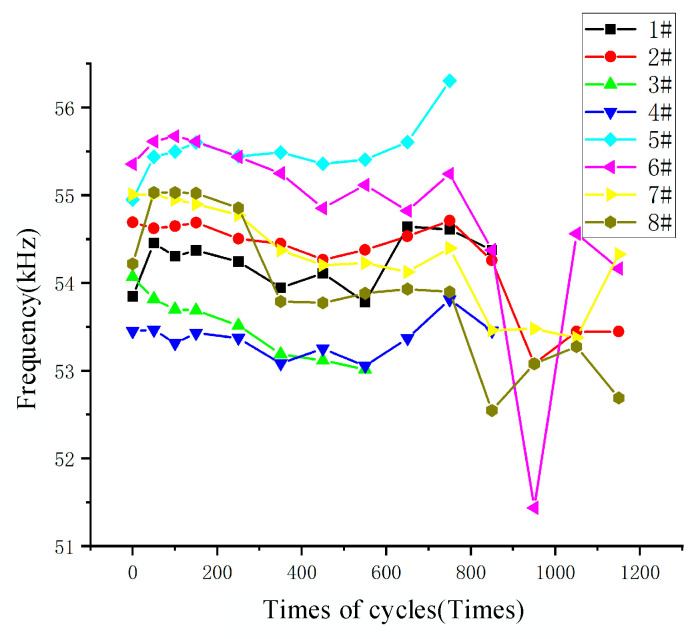
Variations in sample resonant frequency with the number of cycles of temperature cycling experiments.

Figure 14 demonstrates the variation in the resonant frequency of the sample under multiple temperature cycles. The resonance frequency of the samples remained within a range of 53 kHz to 56 kHz throughout the test. During the first 200 thermal cycles, most devices exhibited a slight increase in resonance frequency, with relatively stable overall behavior. Between 200 and 800 cycles, the amplitude of the frequency changes became significantly larger, with several devices failing around 800 cycles. Specifically, samples 1# and 3# failed at 900 cycles, sample 4# at 600 cycles, and sample 5# at 800 cycles. After 1000 cycles, the surviving samples showed a decrease in resonance frequency compared to their initial values, with decreases ranging from 0.533 kHz to 1.6328 kHz. These trends suggest that thermal cycling initially stabilizes the device but gradually introduces stress-related degradation, ultimately leading to mechanical failure in some samples and a frequency decline in others.

**Figure 15:**
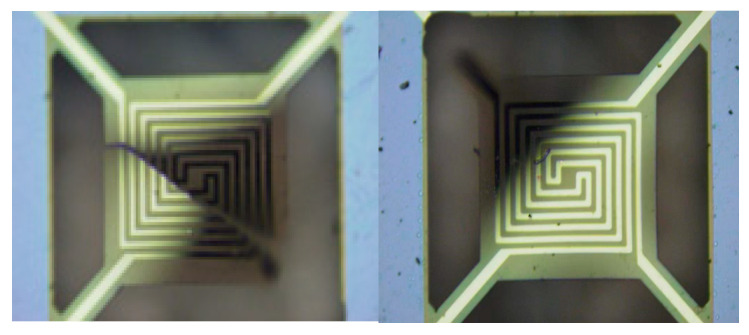
Failure device appearances.

Figure 15 illustrates the failed sample. From the appearance of the failed sample, it can be seen that the main area of failure was the cantilever beam. This was also the part of the stress concentration observed in the simulation.

## 6. Conclusions

The Pirani gauge was used for finite element modeling and analysis to simulate the behavior of the multilayer film structure under various conditions, including 25 °C room temperature, −55 °C low temperature, and 125 °C high temperature. The simulations analyzed the morphological changes and thermal stress of the device under these conditions. The simulation results showed that the magnitude and distribution of thermal stress were closely related to temperature differences, materials, and structure. The greater the temperature difference, the more significant the thermal stress. Specifically, the thermal stress change to silicon dioxide was less affected by temperature, while silicon nitride was more affected, and Pt material was affected the most. Structurally, the clamped-end beam made of Pt material was a stress concentration area, and the inner edge of the silicon substrate also showed significant stress concentration. The top layer of the silicon nitride film could reduce the thermal stress on the Pt material to some extent, providing support and protection. To mitigate these risks, future optimization of MEMS thin film structures should focus on structural design improvements. Specifically, adding chamfers to sharp corners in the cantilever beams can help reduce stress concentrations that may lead to localized failure. Additionally, when incorporating heat dissipation holes, increasing the number of cantilever beams can further enhance structural stability and more evenly distribute thermal stress, thereby reducing the risk of deformation and improving the overall durability of the device.

Theoretically, alternating hot and cold thermal cycling conditions can induce changes in the internal structure of materials by facilitating the movement of dislocations and defects, thus reducing or eliminating residual stress generated during film deposition [21]. Experimental results indicated that the multilayer composite film structure exhibited significant concavity after multiple temperature cycles. Furthermore, the resonance frequency of the multilayer composite film fluctuated significantly during the middle thermal cycles, with both increases and decreases observed, and several samples experienced failure due to structural degradation during this phase. Eventually, the overall trend showed a decrease in resonance frequency. Since the stiffness of the film structure is positively correlated with its resonance frequency [22], this suggests that although temporary increases in stiffness may occur due to internal structural realignment, the overall stiffness of the film gradually decreases over time as a result of the cumulative effects of thermal cycling.

## Figures and Tables

**Table 1:** Sample symbols and definitions.

Heating Resistor	Design Resistance Value (ohm)	Corresponding Interface
Rh1	230	Pad1, Pad2
Rh2	230	Pad3, Pad4

**Table 2:** Main parameters of the material.

Parameters	Si	SiO_2_	Pt	Si_3_N_4_
Young’s modulus (GPa)	160	69	168	250
Poisson’s ratio	0.22	0.35	0.38	0.23
Thermal conductivity(W/(m·K))	150	1.4	71.6	20
Coefficient of thermal expansion (1/K)	2.5 × 10^−6^	0.5 × 10^−6^	8.8 × 10^−6^	2.3 × 10^−6^

## Data Availability

The data that support the findings of this study are available from the corresponding authors upon reasonable request.
